# High-Performance Detection of *Mycobacterium bovis* in Milk Using Recombinase-Aided Amplification–Clustered Regularly Interspaced Short Palindromic Repeat–Cas13a–Lateral Flow Detection

**DOI:** 10.3390/foods13111601

**Published:** 2024-05-21

**Authors:** Jieru Wang, Nan Wang, Lei Xu, Xiaoyu Zeng, Junsheng Cheng, Xiaoqian Zhang, Yinghui Zhang, Dongdong Yin, Jiaojiao Gou, Xiaocheng Pan, Xiaojie Zhu

**Affiliations:** 1 Anhui Province Key Laboratory of Livestock and Poultry Product Safety Engineering, Livestock and Poultry Epidemic Diseases Research Center of Anhui Province, Key Laboratory of Pig Molecular Quantitative Genetics of Anhui Academy of Agricultural Sciences, Institute of Animal Husbandry and Veterinary Sciences, Anhui Academy of Agricultural Sciences, Hefei 230031, China; wangjr0317@163.com (J.W.); jj18395415715@163.com (J.G.); 2 China Institute of Veterinary Drug Control, Beijing 100000, Chinazyh8977@126.com (Y.Z.)

**Keywords:** milk, food safety, bovine tuberculosis, CRISPR–Cas13a, rapid detection

## Abstract

*Mycobacterium bovis* (*M. bovis*), the microorganism responsible for bovine tuberculosis (bTB), is transferred to people by the ingestion of unpasteurized milk and unprocessed fermented milk products obtained from animals with the infection. The identification of *M. bovis* in milk samples is of the utmost importance to successfully prevent zoonotic diseases and maintain food safety. This study presents a comprehensive description of a highly efficient molecular test utilizing recombinase-aided amplification (RPA)–clustered regularly interspaced short palindromic repeat (CRISPR)-associated protein (Cas) 13a–lateral flow detection (LFD) for *M. bovis* detection. In contrast to ELISA, RPA–CRISPR–Cas13a–LFD exhibited greater accuracy and sensitivity in the detection of *M. bovis* in milk, presenting a detection limit of 2 × 10^0^ copies/μL within a 2 h time frame. The two tests exhibited a moderate level of agreement, as shown by a kappa value of 0.452 (95%CI: 0.287–0.617, *p* < 0.001). RPA–CRISPR–Cas13a–LFD holds significant potential as a robust platform for pathogen detection in complex samples, thereby enabling the more dependable regulation of food safety examination, epidemiology research, and medical diagnosis.

## 1. Introduction

Bovine tuberculosis (bTB) is a chronic disorder primarily resulting from *Mycobacterium bovis* (*M. bovis*), a zoonotic pathogen with a global distribution that causes significant economic losses to milk and meat producers [1,2]. The most common source of pathogenic mycobacteria is non-pasteurized milk, particularly in developing nations with a higher prevalence of bTB. Reports have shown the detection of *M. bovis* in milk that has not been properly pasteurized, as well as in milk samples obtained from cattle that do not show a reaction to tuberculin [3,4,5,6]. Tissue specimens (lymph nodes and lungs) and secretions (milk and sputum) can be used in microbiological culture. Even though the microbiological culture approach continues to be the most reliable and widely accepted technique for diagnosing bTB, it may take 4 to 8 weeks to culture the organism. In China, Australia, Japan, England, and Argentina, the National Program for Control and Eradication of bTB requires the use of a tuberculin purified protein derivative (PPD) for bTB diagnosis in animals [7]. However, tuberculin PPD lacks sufficient sensitivity and specificity and has economic disadvantages due to animal immobilization requirements and frequent applications. For early detection, the interferon-γ (IFN-γ) test is used to detect IFN-γ production resulting from lymphocyte stimulation with specific antigens in blood samples. Diagnostic tests based on antibody detection in blood and milk represent another option for *M. bovis* detection in cattle [8]. Enzyme-linked immunosorbent assays (ELISAs) have been utilized. However, these methods require 48–72 h for processing and the specialized training of veterinarians [9]. The antibody lateral flow test targets immunodominant antigens that elicit a humoral response. However, the level of antibodies in the early *M. bovis* infection stage is usually very low, and these antibodies are not detectable with currently available antibody lateral flow tests [10]. The polymerase chain reaction (PCR) serves as a direct test available for detecting *M. bovis* in cattle. However, PCR requires adequately trained personnel along with costly reagents and equipment [11]. Furthermore, calcium may inhibit polymerase activity by competing with magnesium ions present in milk [12]. During an outbreak, if a cow is determined to be negative, there is no suggestion to perform further testing. This might result in the misidentification of animals in the herd as false negatives, which poses a risk to effectively managing the infection. Therefore, improving and simplifying bTB diagnosis has become a priority measure.

The protein (Cas) 13a that is related to clustered regularly interspaced short palindromic repeat (CRISPR) has been produced as a very sensitive diagnostic method for in vitro nucleic acid identification. This is achieved via the non-specific RNA cutting capability of Cas13a [13]. The system relies on RNA fluorescence reporting probes, which are cut to emit fluorescence when Cas13a is activated by target RNA. Due to the powerful RNase activity of Cas13a, a single-target RNA-activated Cas13a can cut thousands of RNA probes to amplify the signal of the target RNA in the reaction system [14]. Furthermore, the sensitivity of the method is greatly enhanced by isothermal amplification, a technique that employs T7 transcription (RNA virus) combined with recombinant polymerase amplification (DNA amplification only) to achieve secondary amplification of the signal [15]. Detection using this approach has been successfully applied for nucleic acids (DNA and RNA), including SARS-CoV-2 [16], pathogenic bacteria [17,18], hepatitis B virus [19], cancer-associated RNA [20], porcine circovirus (PCV) [21], classical swine fever virus (CSF) [22], and *Mycobacterium tuberculosis* [23]. Nevertheless, the utilization of CRISPR–Cas in milk has proven infrequent for the identification of *M. bovis*.

This investigation included the development of a system for detecting *M. bovis* in milk. The system was constructed by integrating CRISPR–Cas13a, recombinase-aided amplification (RPA), and lateral flow detection (LFD). The CRISPR–Cas technique was developed employing the *M. bovis* MPB70 gene as the target. Our approach has the potential to facilitate the rapid identification of bTB in milk.

## 2. Materials and Methods

### 2.1. Bacteria Culture and DNA Extraction

Middlebrook 7H9 liquid medium was employed to facilitate the growing of *Mycobacterium* H37Rv ATCC 25618 (H37Rv), *M. bovis bacille Calmette–Guérin* (BCG) strain Pasteur, *Mycobacterium smegmatis mc^2^155* (Ms), and *Mycobacterium kansasii* ATCC 12478 (*M. kansasii*) for 2 to 4 weeks at 37 °C. Tryptone soya broth (TSB, Thermo Fisher Scientific, Shanghai, China) was utilized to conduct overnight culturing of *Escherichia coli* ATCC 25922 (*E. coli*), *Listeria monocytogenes (L. monocytogenes)* ATCC 51772, *Staphylococcus aureus* CICC 21600 (*S. aureus*), and *Salmonella enterica* subsp. enterica serovar Dublin (*S*. Dublin) at 37 °C. All strains were acquired from the China Institute of Veterinary Drug Control repository. Total DNA from each bacterial strain was obtained utilizing QIAamp BiOstic Bacteremia DNA Kit (QIAGEN, Shanghai, China) following the manufacturer’s instructions.

### 2.2. Recombinase-Aided Amplification (RPA) Primer Sequences and crisprRNA (crRNA) Preparation

The RPA primers were designed based on the nucleotide base sequence of the bTB MPB70 gene. The T7 promoter sequence (Promoter T7: GAAATTAATACGACTCACTATAGGG) was added to the 5′ end of the RPA forward primer. Two Cas13a crRNAs targeting the RPA amplification products of the MPB70 gene were developed. The T7 promoter sequence and universal Cas13a hairpin sequences (GATTTAGACTACCCCAAAAACGAAGGGGACTAAAAC) were sequentially attached to the 5′ ends. The synthetic DNA probe, also known as crRNA, was annealed to double-stranded DNA (dsDNA) utilizing an annealing buffer specifically designed for DNA oligos (Beyotime, Shanghai, China) and purified by gel extraction. The HiScribe T7 Quick High Yield RNA synthesis kit (NEB, Ipswich, MA, USA) was employed for the transcription of dsDNA to crRNA following the directions provided by the manufacturer. The dsDNA was transcribed to crRNA using the HiScribe T7 Quick High Yield RNA synthesis kit (NEB, Massachusetts, USA), and the Agencourt RNAClean XP kit (Beckman Coulter, Brea, CA, USA) was employed for the purification of crRNA. The lateral flow RNA reporter probe was 5′-/56-FAM/mArArUrGrGrCmAmArArUrGrGrCmA/3Bio/-3′ and the qPCR RNA reporter probe was 5′-/56-FAM/TrCrArUrUrUrG/3TAMRA/-3′. The primers and sequences used in this study were manufactured by the General Biological System Co. (Anhui, China) (Table 1).

### 2.3. Preparation of Recombinant pUC57-MPB70 Plasmid

The recombinant pUC57-MPB70 plasmid was constructed according to our previously reported method [21]. Briefly, the sequence of the MPB70 gene was amplified from *M. bovis* CVCC 68001 (stored in the China Institute of Veterinary Drug Control repository) [24] by PCR using MPB70-F1 (GGTCAGTACACGGTGTTCGC) and MPB70-R1 (TACGCCGGAGGCATTAGC) and digested by BamHΙ and Sall. The digested PCR fragment was cloned into a pUC57 plasmid (Takara, Dalian, China) previously digested with the same enzyme and recovered with T4 DNA ligase. Then, the pUC57-MPB70 plasmid was transformed into DH5a and plasmid was extracted. Subsequently, the enzyme-digested products were identified by electrophoresis and sequencing. Qubit dsDNA HS assay kits (Thermo Fisher Scientific, China) were employed to determine the recombinant plasmid concentration, and the DNA amount was calculated.

### 2.4. RPA Reactions

An RPA kit (DNA-LS01, LeSunBio, Wuxi, China) was utilized to conduct one-step RPA reactions to amplify target DNA following the manufacturer’s directions. Under isothermal conditions (37 °C), the target gene fragment was amplified for 40 min using the sample DNA as the template. Briefly, the reaction (50 µL) consisted of 1–5 μL DNA, 25 μL RPA polymerase amplification buffer, 240 pM MPB70-RPA primers, 2.5 μL magnesium acetate, and ddH_2_O. The optimum primers were selected using the Bioptic Qsep 100 automatic nucleic acid analysis system. Afterward, the products of the RPA reaction were transferred to the CRISPR–Cas13a cleavage assay.

### 2.5. RPA–CRISPR–Cas13a–LFD

For each CRISPR–Cas13a-based assay, the reaction (9 μL) consisted of 22.5 nM crRNA probe, 45 nM LwaCas13a (Magiltd, Hefei, China), 0.25 μL RNase inhibitor, 125 nM lateral flow RNA reporter probe, 1 mM dNTP, 0.4 μL T7 RNA polymerase mix (NEB, USA), 1 μL RPA product, and buffer. RPA–CRISPR–Cas13a nucleic acid identification was conducted in a water bath for 40 min at 37 °C. Hybridetect assay buffer (Magiltd) was utilized to conduct a 10-fold dilution of the product, which was loaded onto LFD strips (Magigen, Guangzhou, China). Following the incubation for 3–5 min, the outcomes were documented.

### 2.6. RPA–CRISPR–Cas13a–Quantitative Real-Time PCR (qPCR)

The RPA–CRISPR–Cas13a nucleic acid identification system (the 9 μL reaction: 22.5 nM crRNA probe, 45 nM LwaCas13a, 0.25 μL RNase inhibitor, 125 nM qPCR RNA reporter probe, 1 mM dNTP, 0.4 μL T7 RNA polymerase mix, and 1 μL RPA product) was incubated at 37 °C on the QuantStudio^TM^ 6 Flex real-time quantitative fluorescence PCR instrument for 40–90 min, and fluorescence signals based on qPCR RNA reporter probes were collected once per minute.

### 2.7. Analytical Sensitivity and Specificity of RPA–CRISPR–Cas13a–LFD

The pUC57-MPB70 was prepared as follows. Briefly, the pUC57-MPB70 copy number was calculated based on the following formula:

(1) $$Number\;of\;copies=\frac{Amount\;of\;DNA\;\left(ng\right)\times Avogadro^{\prime }s\;constant}{Length\;of\;DNA\;\left(bp\right)\times conversion\;factor\times Average\;mass\;of\;1\;bp\;of\;dsDNA}$$

Avogadro’s constant—this number (6.02 × 10^23^) represents the number of molecules in 1 mole. Conversion factor—the conversion factor (1 × 10^9^) is required to convert the value to ng. The average mass of 1 bp of dsDNA—the average mass of 1 bp of dsDNA is 660 g/mole. The concentration of pUC57-MPB70 was detected using Qubit dsDNA HS Assay Kits (Thermo Fisher Scientific, China), and then the amount of DNA was calculated. Length of DNA (3292 bp) is the length of pUC57-MPB70 (2710 bp) plus the length of MPB70 (582 bp). Then, the pUC57-MPB70 plasmid (copy number was 2 × 10^10^) was diluted 10-fold in pasteurized whole cow milk from local markets. The dilutions were then used to create a standard curve for RPA reactions, taking into account the varied molecular weights. Afterwards, the reaction products were subjected to CRISPR–Cas13a–LFD. The analytical sensitivity was calculated employing the data. The genomic DNAs from a panel of pathogens were employed in pasteurized milk to evaluate the analytical specificity, including BCG, H37Rv, Ms, *M. kansasii*, *E. coli*, *S. aureus*, *L. monocytogenes*, and *S.* Dublin.

### 2.8. Preparation of Milk Samples and DNA Extraction

Milk samples were obtained from 114 milking cows from 10 dairy herds in Anhui province (China). We prepared the milk samples as previously reported [23]. Briefly, samples of 4–7 mL milk underwent 15 min centrifugation at 4696× *g* at 4 °C. The supernatant was discarded, and the fat was eliminated using a sterile cotton swab. Subsequently, 2 mL of 0.5 M EDTA was added with repeated blowing and beating of the sediment solution and underwent 5 min centrifugation at 14,000× *g*. Finally, the precipitate was resuspended in 800 µL 4% NaOH and subjected to 10 min of inactivation. Then, the mix was introduced to a tube of DNeasy Blood & Tissue kit (QIAGEN, Shanghai, China) for DNA extraction. The extracted DNA samples were used in bTB-specific RPA–CRISPR–Cas13a–LFD and –qPCR.

### 2.9. Milk ELISA

Depending on a recombinant chimera of four specific proteins, CFP10/ESAT-6/MPB70/MPB83, as the antigen, we used milk ELISA to determine *M. bovis* (Wuhan Keqian Biology Co., Ltd., Wuhan, China) based on our previous study [10]. We used an undiluted milk sample. The manufacturer’s threshold was set at 0.1. Measurements of optical density (OD) were conducted at 450 nm. The sample-to-positive ratio value was calculated for each sample as (OD_sample_ − OD_Negative_)/(OD_Postive_ − OD_Negative_). A sample was positive when the milk ELISA of the sample-to-positive ratio percentage was >10%.

### 2.10. Data Analysis

Cohen’s kappa test statistic was employed to assess the agreement level between RPA–CRISPR–Cas13a–LFD and milk ELISA [25].The Cas13a–qPCR and milk ELISA were expressed as the mean of ≥3 independent reactions ± SD. Data panels were generated using GraphPad Prism 9 to determine the means and SDs to calculate the analysis of variance (ANOVA). The paired *t*-test was used to detect mean differences in quantification.

## 3. Results

### 3.1. Optimizing the Primers for CRISPR–Cas13a-Based bTB Detection Platform

**Figure 1:**
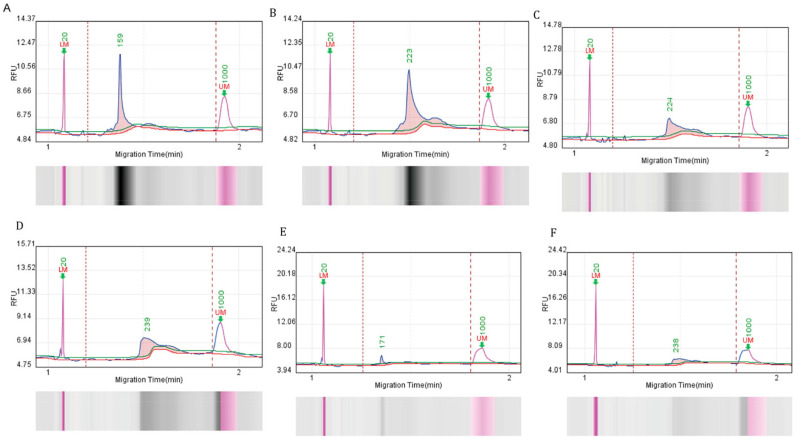
Capillary electrophoresis of RPA-amplified products, corresponding to five primer pairs. (**A**–**F**). Products were analyzed using the Q-sep 100 automatic nucleic acid analysis system. The abscissa axis represents DNA fragment migration time, and the ordinate axis represents fluorescence signal intensity. The sample concentration was calculated by comparing the peak plot area of the standard concentration with the peak plot area of the test sample. Six primer pairs were used for RPA amplification.

The fragment sizes and levels were measured employing a system of Bioptic Qsep 100 automated nucleic acid analysis. The detection peak of six pairs of primer amplification products is shown in Figure 1. The shape of the primer six-peak pattern is smeared rather than unimodal. Afterwards, we conducted a comparison of the sizes of the corresponding RPA amplification products and discovered that they closely matched the sizes that were predicted (Table 2). Therefore, MPB70 primers 1–6 were selected for further assessment.

### 3.2. Identification of High-Activity crRNAs for bTB Detection

**Figure 2:**
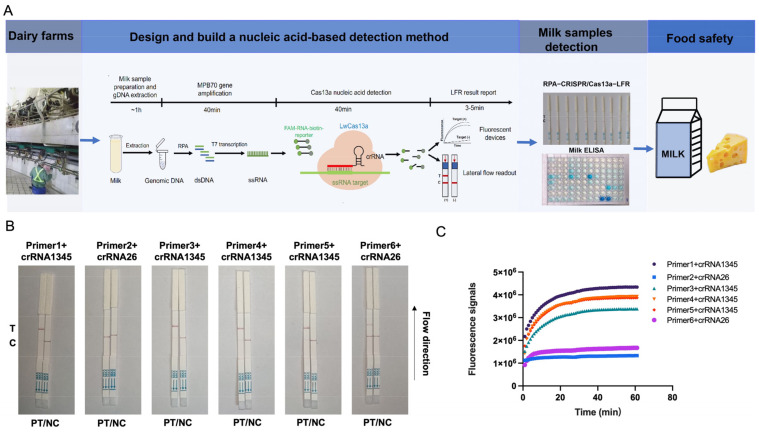
Development of RPA–CRISPR–Cas13a–LFD. (**A**) Schematic diagram showing the Cas13a bTB test workflow. The *M. bovis* MPB70 gene was extracted from milk samples, amplified by RPA, and transcribed to ssRNA to activate the Cas13a nuclease. The nuclease recognized crRNA and cut off the reporter molecule. The reporter molecule appeared as a band on the test strip. RPA = recombinase-aided amplification; ssRNA = single-stranded RNA. (**B**,**C**) Analysis of different crRNAs by RPA–CRISPR–Cas13a–LFD (**B**) and RPA–CRISPR–Cas13a–qPCR (**C**). PT = pUC57-MPB70 standard plasmid; NC = negative control; T = test line; C = control line.

To select the suitable crRNAs for bTB identification, two crRNAs were designed (Table 1) and evaluated using RPA–CRISPR–Cas13a–LFD or –qPCR. The recombinant pUC57-MPB70 plasmid was undetected with primer 2-crRNA2+6 in LFD (Figure 2A) and qPCR (Figure 2B). The detection results with the primer 6-crRNA2+6 test strip were light (Figure 2A) and the fluorescence was slightly increased (Figure 2B). Therefore, based on the results of the LFD and qPCR, primer 1-crRNA1345 was selected to perform RPA–CRISPR–Cas13a–LFD.

### 3.3. Establishment of CRISPR–Cas13a Combined with RPA Amplification for Detection of bTB

**Figure 3:**
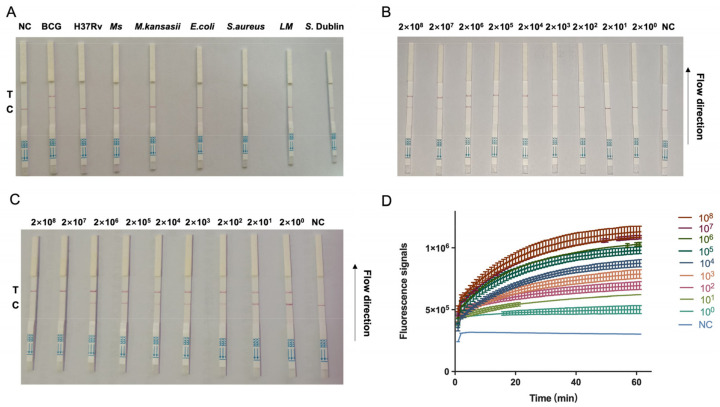
RPA–CRISPR–Cas13a–LFD specificity and sensitivity assays. A 10-fold serial dilution of pUC57-MPB70 was used as the detection template. (**A**) *Mycobacterium bovis bacille Calmette–Guérin (BCG) strain Pasteur*, *Mycobacterium H37Rv* ATCC 25618 (*H37Rv*), *Mycobacterium smegmatis mc^2^155* (*Ms*) and *Mycobacterium kansasii* ATCC 12478 (*M. kansasii*), *Escherichia coli* ATCC 25922 (*E. coli*), *Listeria monocytogenes* ATCC 51772 (*L. monocytogenes*), *Staphylococcus aureus* CICC 21600 (*S. aureus*), *Klebsiella strain* AHKV-S01 (GenBank accession number: CP047360), and *Salmonella* Dublin CICC 21497 (*S.* Dublin). (**B**,**C**) Sensitivity testing of different volumes of DNA: 1 μL (**B**) and 5 μL (**C**). Detection reactions were performed using the following detection limits: 2 × 10^8^ and 2 × 10^0^ copies/µL. (**D**) Sensitivity testing of 5 μL DNA by RPA–CRISPR–Cas13a–qPCR. NC = negative control; T = test line; C = control line.

The combination of CRISPR–Cas13a and RPA amplification was employed to enhance the on-site identification of bTB. Figure 3A shows that only the crRNA targeting the *M. bovis* BCG showed positive signals in the LFD, while for all the other pathogens, negative signals were detected. The combination of CRISPR–Cas13a with RPA amplification and LFD enables the precise detection of bTB. In order to assess the level of detection sensitivity of the combined CRISPR–Cas13a and RPA amplification, we diluted the pUC57-MPB70 plasmid DNA (1 μL) from a concentration of 2 × 10^8^ to 2 × 10^0^ copies/μL then utilized it as a template. A comparison was conducted between the detection sensitivity of the RPA and Cas13a cleavage assays. This study revealed that the limit of the amount of bTB MPB70 gene that can be detected by RPA was 2 × 10^2^ copies/μL (Figure 3B). Furthermore, in order to enhance the amplification effectiveness of RPA, the volume of the DNA template was raised to 5 μL. The RPA–CRISPR–Cas13a–LFD or –qPCR findings demonstrated that the improved RPA assay for the bTB MPB70 gene had a limit of detection of 2 × 10^0^ copies/μL. This was determined by employing a positive plasmid with MPB70 as the amplification template (Figure 3C,D). Thus, the combination of RPA amplification with the very potent crRNA enhances the suitability of CRISPR–Cas13a for the sensitive identification of bTB in the field.

### 3.4. Detection of bTB in Clinical Milk Samples by RPA–CRISPR–Cas13a–LFD

To evaluate the reliability of RPA–CRISPR–Cas13a–LFD for bTB detection in milk samples, we used 114 bTB-positive and -negative samples (Figure S1) and validated the results using ELISA. Table 3 shows that 80 samples (70.2%, 80/114) tested positive in the RPA–CRISPR–Cas13a–LFD. Fifty-nine samples (51.7%, 59/114) tested positive both in the RPA–CRISPR–Cas13a–LFD and milk ELISA. Twenty-one samples (18.4%, 21/114) tested positive in the RPA–CRISPR–Cas13a–LFD only. The agreement of the test outcomes between the RPA–CRISPR–Cas13a–LFD and milk ELISA is illustrated. Generally, 67 of 114 samples tested positive in the milk ELISA when 0.1 was used as the cut-off value for the assay. The overall agreement for the positive and negative samples between the RPA–CRISPR–Cas13a–LFD and milk ELISA was 74.6% (85/114). There was a moderate level of agreement between these two tests (kappa = 0.452, 95%CI: 0.287–0.617, *p* < 0.001).

## 4. Discussion

The consumption of unpasteurized milk and dairy products prepared with raw milk, the regular contact between humans and animals carrying infections, and insufficient disease control measures are risk factors of zoonotic tuberculosis. In addition to inhaling aerosols or the direct contact with mucous membranes and skin abrasions, the primary mode of transmission of *M. bovis* to humans is via contaminated foods, primarily unpasteurized dairy products or, to a lesser extent, untreated meat products [6]. The intermittent excretion of mycobacteria in milk enables up to 30% of infected cows to eliminate mycobacteria through milk [3,26]. In Tanzania, despite considerable testing and the removal of infected cattle, a residual risk of exposure via milk consumption remains due to the inadequate sensitivity of the skin test [1]. Therefore, it is important to utilize highly sensitive detection methods to improve the *M. bovis* detection rate in raw milk. PCR techniques associated with restriction enzyme analysis patterns, multiplex PCR, nested PCR, and ELISA have been used [3,10,27,28]. In Zambia, *M. bovis* was detected by PCR in the milk of 16 tuberculin-positive cows, and three positive samples (18.7%) were identified by phenotypic methods [3]. In Turkey, 35 raw milk samples were evaluated for the presence of mycobacteria using phenotypic methods and confirmed by PCR–restriction pattern analysis [27]. Our study presents a new approach for detecting nucleic acids depending on CRISPR–Cas13a for bTB detection. To identify a suitable target region, the conserved MBP70 gene of *M. bovis* was selected for RPA primers and crRNA design. The Cas13a identification method elevated the specificity and sensitivity of detecting bTB with a 2 copies/μL detection limit when 5 μL of DNA was used. A previous study reported detection limits of RT-qPCR and dLAMP of 31.61 and 3.88 copies/reaction, respectively [29]. The sensitivity was greater in our method than in RT-qPCR and dLAMP [30].

In this study, there was a moderate agreement between the RPA–CRISPR–Cas13a–LFD and milk ELISA. Twenty-one positive samples detected via RPA–CRISPR–Cas13a–LFD were not detected via milk ELISA. This may be due to the RPA–CRISPR–Cas13a–LFD test being based on the nucleic acid of *M. bovis*, while the antibody ELISA targets immunodominant antigens that elicit a humoral response. During the early phase of infection, the concentration of antibodies is often minimal, and these antibodies are undetectable via milk ELISA. Antibody-based TB screening could be utilized for disease monitoring in animals that have reached the more advanced phases of the illness [31]. However, nucleic acid-based RPA–CRISPR–Cas13a–LFD tests could be employed for diagnosis and long-term monitoring.

Our study had a few limitations. First, we used a milk ELISA to evaluate the accuracy of the RPA–CRISPR–Cas13a–LFD test. Employing highly reliable ways to determine infection status has great potential for future studies. Second, because of the widespread use of blood ELISAs in *M. bovis* dentification, it is imperative to compare its clinical application exclusively with blood ELISAs. Third, the presence of calcium ions in milk samples may affect the PCR outcomes. Therefore, we did not compare the RPA–CRISPR–Cas13a–LFD outcomes with the PCR results. Future studies should prioritize the use of RPA–CRISPR–Cas13a–LFD in combination with primary screening tests on farms, followed by IFN-γ for confirmation.

In conclusion, RPA–CRISPR–Cas13a–LFD has several distinguishing characteristics. First, the method is versatile by allowing for a visual readout through lateral flow detection and by enabling the analysis of samples through fluorescence detection. Consequently, RPA–CRISPR–Cas13a–LFD can be effectively employed in both field and laboratory settings. Second, the detection process is specifically designed and performed at 37 °C, rendering it applicable in under-equipped laboratories or field environments. Therefore, a visual approach for detecting nucleic acids using CRISPR–Cas13a was developed for bTB. Our developed technique could offer another tool for bTB detection as a robust platform for pathogen detection in intricate samples, thereby furnishing more dependable guidance for food safety testing, epidemiology research, and medical diagnosis.

## Figures and Tables

**Table 1:** Recombinase-aided amplification (RPA) primer sequences.

Name	Sequence (5′-3′)
**MPB70-RPA-F1** **MPB70-RPA-R1**	TGTCGGGCCAGCTCAATCCGCAAGTAAA GGATGCTGGTCAGCAGTGACGAATTGGTCTT
**MPB70-RPA-F2** **MPB70-RPA-R2**	CTGCTGACCAGCATCCTGACCTACCACGTA CGCCGGAGGCATTAGCACGCTGTCAATCA
**MPB70-RPA-F3** **MPB70-RPA-R3**	ACGGCTGCACTGTCGGGCCAGCTCAATC GGTGCCGACGACGTTGGCCGGGCTGGTTT
**MPB70-RPA-F4** **MPB70-RPA-R4**	CAGGACCCGGTCGCGGTGGCGGCCTCGAACAAT GGTCAGGATGCTGGTCAGCAGTGACGAATT
**MPB70-RPA-F5** **MPB70-RPA-R5**	AGCTCAATCCGCAAGTAAACCTGGTGGACA CCACTACGTGGTAGGTCAGGATGCTGGTCA
**MPB70-RPA-F6** **MPB70-RPA-R6**	AGCTCAAGACCAATTCGTCACTGCTGACCAG ATTAGCACGCTGTCAATCATGTACACCGTCG
**crRNA1345**	AACACCGTGTACTGACCGCTGTTGAGGG
**crRNA26**	GGCGTTACCGACCTTGAGGCTGTTACCC

**Table 2:** Amplified products as detected by the Bioptic Qsep 100 assay.

Primer Name	Peak Fragment Size (bp)	Theoretical Fragment Size (bp)	Product Concentration (ng/µL)
MPB70 Primer1	159	154	2.82
MPB70 Primer2	223	213	3.91
MPB70 Primer3	224	217	1.99
MPB70 Primer4	239	219	2.3
MPB70 Primer5	171	162	1.08
MPB70 Primer6	238	223	1.42

**Table 3:** Agreement between RPA–CRISPR–Cas13a–LFD assay and milk ELISA (n = 114).

		RPA–CRISPR–Cas13a–LFD		Total
		+ve	−ve	
ELISA	+ve	59	8	67
	−ve	21	26	47
	Total	80	34	114
Kappa (95%CI)	0.452 (95%CI: 0.287–0.617)			

## Data Availability

The original contributions presented in the study are included in the article/Supplementary Material, further inquiries can be directed to the corresponding author.

## Supplementary Materials

The following supporting information can be downloaded at: https://www.mdpi.com/article/10.3390/foods13111601/s1, Figure S1: Clinical sample detection by RPA-CRISPR–Cas13a-LFD. A total of 114 milk samples were used. The red rectangle represents negative results. NC = negative control; T = test line; C = control line.
