# Supplementary material for: High-Performance Detection of Mycobacterium bovis in Milk Using Recombinase-Aided Amplification–Clustered Regularly Interspaced Short Palindromic Repeat–Cas13a–Lateral Flow Detection
Source: Foods. 2024 May 21;13(11):1601. doi: 10.3390/foods13111601 (PMC11171503; doi:10.3390/foods13111601)
Supplement: Supplementary file 1 [file foods-13-01601-s001.zip › foods-2947378-supplementary/Figure S1.pdf]

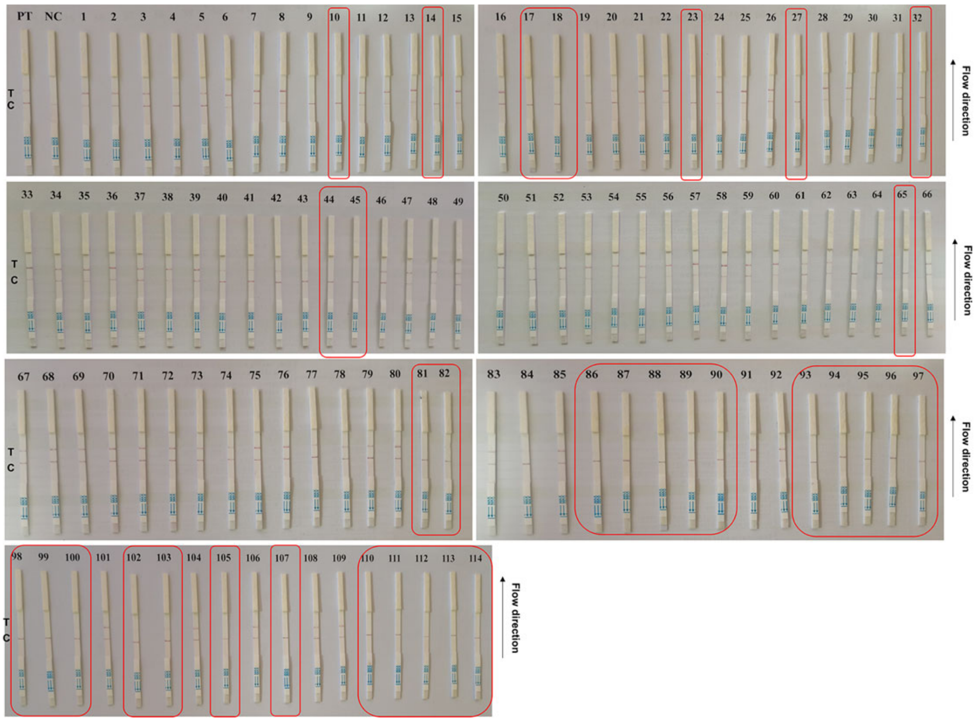


**Figure S1.** Clinical sample detection by RPA-CRISPR–Cas13a-LFD. A total of 114 milk samples were used. The red rectangle represents negative results. NC = negative control; T = test line; C = control line.
