# Supplementary figures and images for: High-Performance Detection of Mycobacterium bovis in Milk Using Recombinase-Aided Amplification–Clustered Regularly Interspaced Short Palindromic Repeat–Cas13a–Lateral Flow Detection
Source: Foods. 2024 May 21;13(11):1601. doi: 10.3390/foods13111601 (PMC11171503; doi:10.3390/foods13111601)

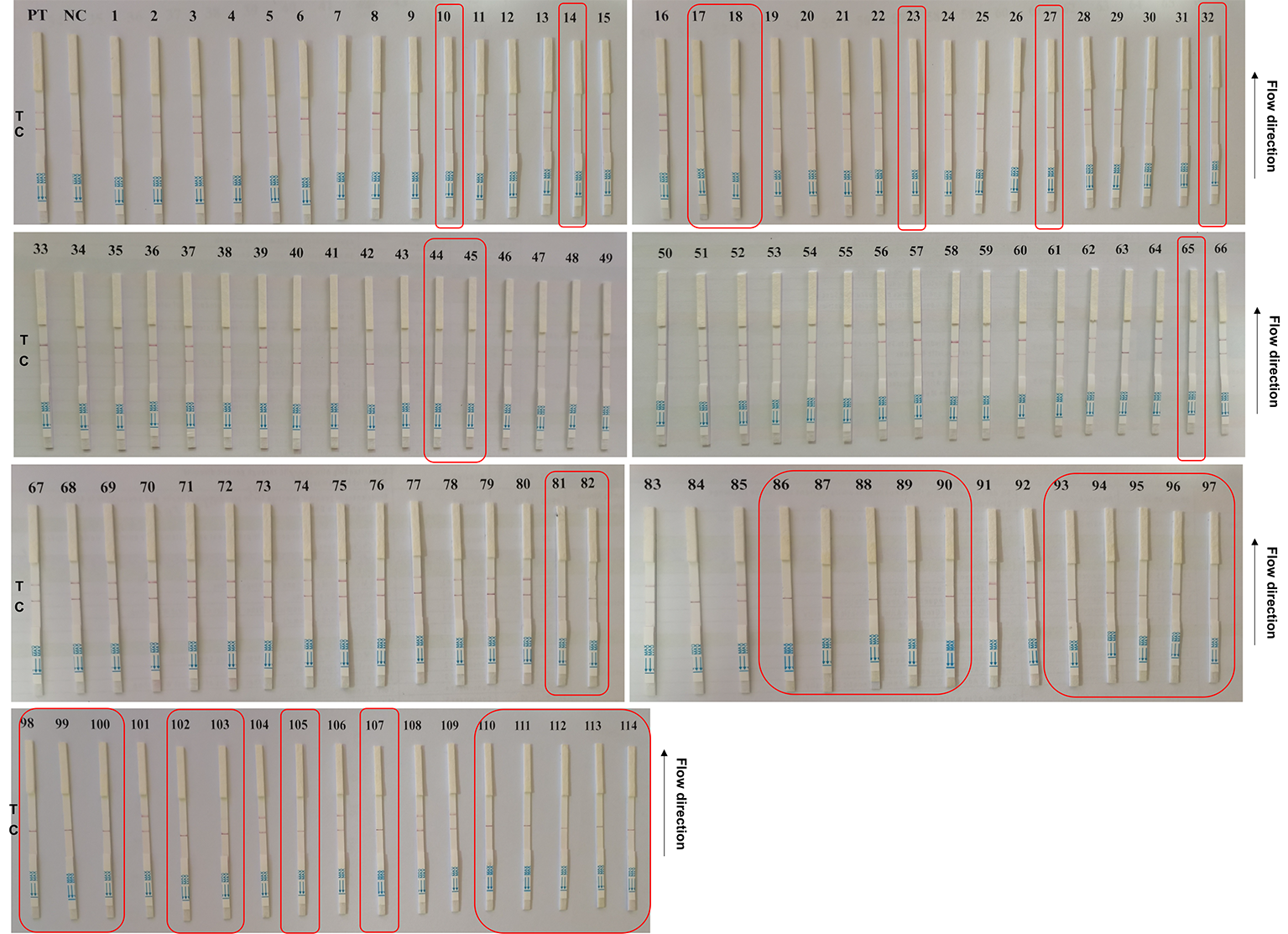

Supplement: Supplementary file 1 [file foods-13-01601-s001.zip › foods-2947378-supplementary/Figure S1.tif]
